# Supplementary material for: The effect of human immunodeficiency virus infection on adverse events during treatment of drug-resistant tuberculosis: A systematic review and meta-analysis
Source: PLoS One. 2021 Mar 4;16(3):e0248017. doi: 10.1371/journal.pone.0248017 (PMC7932087; doi:10.1371/journal.pone.0248017)
Supplement: S1 File — (DOCX) [file pone.0248017.s001.docx]

**The effect of human immunodeficiency virus infection on adverse events during treatment of drug-resistant tuberculosis: a systematic review and meta-analysis**

Gilbert Lazarus^1,*^, Kevin Tjoa^1^, Anthony William Brian Iskandar^1^, Melva Louisa^2^, Evans Sagwa^3^, Nesri Padayatchi^4^, Vivian Soetikno^2,*^

^1^Faculty of Medicine, Universitas Indonesia, Jakarta 10430, Indonesia

^2^Department of Pharmacology and Therapeutic, Faculty of Medicine, Universitas Indonesia, Jakarta 10430, Indonesia

^3^Independent Pharmacoepidemiologist, Windhoek, Namibia and Nairobi, Kenya.

^4^CAPRISA MRC-HIV-TB Pathogenesis and Treatment Research Unit, Durban, South Africa

***Corresponding authors:**

Gilbert Lazarus, Faculty of Medicine, Universitas Indonesia, Jl. Salemba Raya No. 6, Jakarta 10430, Indonesia, Indonesia. E-mail: [gilbert.lazarus@ui.ac.id](mailto:gilbert.lazarus@ui.ac.id)

Vivian Soetikno, MD, Department of Pharmacology and Therapeutic, Faculty of Medicine, Universitas Indonesia, Jl. Salemba Raya No. 6, Jakarta 10430, Indonesia. E-mail: [vivian.soetikno@ui.ac.id](mailto:vivian.soetikno@ui.ac.id)

**Table and Contents**

| Supplementary Material | Page |
| --- | --- |
| Methods | [4](#TableS1_rev) |
| S1 Table. Deviations from the protocol | [4](#TableS1) |
| Search strategy | [5](#Searchstrategy) |
| S2 Table. Literature search strategy | [5](#TableS1) |
| Study eligibility criteria | [8](#Eligcrit) |
| S3 Table. PICOTS framework | [8](#TableS2) |
| Data extraction and quality assessment | [8](#Data) |
| Definition of drug-resistant tuberculosis (DR-TB) | [8](#DRTB) |
| Quality assessments | [9](#Qual) |
| S4 Table. Signaling questions for methodological quality assessments of cohort studies using the Newcastle-Ottawa Scale | [9](#TableS3) |
| S5 Table. Signaling questions for methodological quality assessments of cross-sectional studies using the modified Newcastle-Ottawa Scale | [10](#TableS4) |
| Statistical analysis | [12](#Statanalysis) |
| Results | [13](#Results) |
| S6 Table. Characteristics of the included studies | [13](#TableS5) |
| S7 Table. Results of quality assessment of included cohort studies | [21](#TableS6) |
| S8 Table. Results of quality assessment of included cross-sectional studies | [23](#TableS7) |
| S1 Fig. Results of risk of bias assessment of included non-randomized studies of intervention. | [24](#FigS1_rev) |
| S9 Table. Study-specific outcomes on the risk of developing adverse events | [25](#TableS8) |
| S2 Fig. Pooled unadjusted effects on the association between HIV co-infection and the occurrence of adverse events | [28](#FigS1) |
| S3 Fig. Results of sensitivity analysis for the pooled adjusted effects on the association between HIV co-infection and adverse events occurrence. | [28](#FIgS2) |
| S4 Fig. Results of subgroup analyses of the pooled unadjusted effects on the association between HIV co-infection and adverse events occurrence | [29](#FigS3) |
| S5 Fig. Contour-enhanced funnel plot with pseudo 95% confidence interval indicating no publication bias (as shown by symmetry) for the pooled adjusted effects on the association between HIV co-infection and adverse events occurrence | [30](#FigS4) |
| S10 Table. Summary of meta-analysis on specific AE outcomes | [31](#TableS9) |
| References | [33](#References) |

**METHODS**

**S1 Table.** Deviations from the protocol

| No | Explanation |
| --- | --- |
| 1 | In the initial protocol, we specified odds ratios (ORs) as the common effect measure in our meta-analysis. However, we discovered that the included studies utilized different effect measures i.e., ORs, relative risks (RRs), and hazard ratios (HRs). To the best of our knowledge, there is currently no known method to convert OR to HR and vice versa. Furthermore, we believe that it is not appropriate to pool studies utilizing OR, RR, and HR into a single model as it may introduce substantial heterogeneity, especially when the outcome is more common as OR always overestimates RR. As we have proven in the ‘Statistical analysis’ section, the pooling of HRs and RRs in a single model may introduce bias ratios by a factor of 1.80, 2.47, and 19.00 for outcome probabilities between 0.2-0.8, 0.1-0.9, and 0.05-0.95, respectively.[1] Hence, we decided to choose RR as the common effect measure and convert HR/OR to RR. This decision was also made as most of the included studies in our systematic review were longitudinal studies. However, the pooling of these converted figures may still introduce heterogeneity to our model as bias ratios by a factor of 1.16, 1.45, and 1.93 for outcome probabilities between 0.2-0.8, 0.1-0.9, and 0.05-0.95 were still present. Therefore, we decided to perform a subgroup analysis based on the original effect size type. The subgroup analysis revealed negligible heterogeneity for both subgroups (HR: I^2^=0%, p=0.47; OR: I^2^=0%, p=0.82). Furthermore, our primary analysis also indicated negligible heterogeneity (I^2^=0%; p=0.75), thus rendering these conversions worthwhile. |
| 2 | Initially, we intended to assess potential publication bias using the Doi plot and Luis Furuya-Kanamori (LFK) index, which was proposed by Furuya-Kanamori et al as a substitute of funnel plot.[2] However, referring to the Cochrane Handbook[3], funnel plot is still the recommended tool to assess potential publication bias. [4] Furthermore, to increase the sensitivity of the assessment, we utilized a contour-enhanced funnel plot to investigate potential sources of publication bias.[5] We also utilized Egger’s[6] and Begg’s[7] tests to complement the visual inspection. In the case of evident publication bias, we performed a trim-and-fill analysis to adjust for potential non-reporting biases.[8] In light of this, we decided to perform data analyses (i.e., meta-analyses and data visualization) with R ver. 4.0.0 (R Foundation for Statistical Computing, Vienna, Austria)[9] with the additional *meta* (ver. 4.9-6)[10]*, metafor* (ver. 1.4-0)[11], and *robvis*[12] packages, while additional analyses were performed with MetaXL software ver 5.3. ([www.epigear.com](http://www.epigear.com))[13]. |
| 3 | We initially intended to analyze our findings with the Mantel-Haenszel fixed-effect methods. However, as stated in the systematic review guideline by Riley et al., a systematic review of prognostic factor studies is likely to yield unexplained heterogeneity due to various factors including patients’ characteristics and differences in interventions received and length of follow-up.[14] Hence, we decided to pool our results using the DerSimonian-Laird inverse variance random-effect methods. |
| 4 | We realized that it may be inappropriate to indifferently pool the adjusted outcomes of each study into a single model as the adjustment factors utilized may substantially differ, thus subsequently complicating the interpretation of our meta-analysis results[14]. Hence, we decided to set an additional inclusion criterion to the meta-analysis, in which a study must adjust for at least age and sex to be included in the quantitative analysis of adjusted outcomes. |

**Search strategy**

**S2 Table.** Literature search strategy

| Database | Keywords |
| --- | --- |
| PubMed | ("second line anti-tuberculosis" OR "levofloxacin"[MeSH Terms] OR "moxifloxacin"[MeSH Terms] OR "bedaquiline" OR "linezolid"[MeSH Terms] OR "clofazimine"[MeSH Terms] OR "cycloserine"[MeSH Terms] OR "terizidone" OR "ethambutol"[MeSH Terms] OR "pyrazinamide"[MeSH Terms] OR "delamanid" OR "meropenem"[MeSH Terms] OR "imipenem"[MeSH Terms] OR "streptomycin"[MeSH Terms] OR "amikacin"[MeSH Terms] OR "ethionamide"[MeSH Terms] OR "prothionamide"[MeSH Terms] OR "para-aminosalicylic acid" OR "kanamycin"[MeSH Terms] OR "capreomycin"[MeSH Terms] OR "amoxicillin potassium clavulanate combination"[MeSH Terms] OR "thioacetazone"[MeSH Terms] OR "clarithromycin"[MeSH Terms] OR "high-dose isoniazid" OR "imipenem/cilastatin") AND ("tuberculosis, multidrug resistant"[MeSH Terms] OR "MDR-TB" OR "multi-drug resistant tuberculosis" OR "multidrug resistant tuberculosis") AND ("adverse events" OR "adverse drug reaction reporting systems"[MeSH Terms] OR "adverse effects" OR "adverse drug reaction" OR "safety"[MeSH Terms] OR "tolerability" OR "side effect" OR "toxicity" OR "chemically induced disorders"[MeSH Terms] OR "poisoning"[MeSH Terms]) AND ("human immunodeficiency virus proteins"[MeSH Terms] OR "acquired immunodeficiency syndrome"[MeSH Terms] OR "hiv"[MeSH Terms] OR "human immunodeficiency virus" OR "immunosuppression" OR "acquired immunodeficiency syndrome" OR "HIV") |
| CENTRAL | #1 MeSH descriptor: [Tuberculosis, Multidrug-Resistant] explode all trees  #2 MeSH descriptor: [Drug-Related Side Effects and Adverse Reactions]  explode all trees  #3 MeSH descriptor: [Chemically-Induced Disorders] explode all trees  #4 MeSH descriptor: [Safety] explode all trees  #5 MeSH descriptor: [Drug Tolerance] explode all trees  #6 MeSH descriptor: [Adverse Drug Reaction Reporting Systems] explode  all trees  #7 MeSH descriptor: [Poisoning] explode all trees  #8 MeSH descriptor: [Fluoroquinolones] explode all trees  #9 MeSH descriptor: [Linezolid] explode all trees  #10 MeSH descriptor: [Clofazimine] explode all trees  #11 MeSH descriptor: [Cycloserine] explode all trees  #12 MeSH descriptor: [Ethambutol] explode all trees  #13 MeSH descriptor: [Pyrazinamide] explode all trees  #14 MeSH descriptor: [Carbapenems] explode all trees  #15 MeSH descriptor: [Streptomycin] explode all trees  #16 MeSH descriptor: [Kanamycin] explode all trees  #17 MeSH descriptor: [Isonicotinic Acids] explode all trees  #18 MeSH descriptor: [Capreomycin] explode all trees  #19 MeSH descriptor: [Thioacetazone] explode all trees  #20 MeSH descriptor: [Amoxicillin-Potassium Clavulanate Combination]  explode all trees  #21 MeSH descriptor: [Clarithromycin] explode all trees  #22 MeSH descriptor: [Cilastatin, Imipenem Drug Combination] explode all  trees  #23 #1 OR "multi-drug resistant tuberculosis" OR "MDR-TB" OR "multidrug resistant tuberculosis" OR "drug resistant tuberculosis"  #24 {OR #2-#7} OR "adverse events" OR "adverse effects" OR "adverse drug reaction" OR "side effect"  #25 {OR #8-#22} OR "second-line anti-tuberculosis" OR "bedaquiline" OR "terizidone" OR "delamanid" OR "para-aminosalicylic acid" OR "high-dose isoniazid"  #26 MeSH descriptor: [HIV] explode all trees  #27 MeSH descriptor: [HIV Infection] explode all trees  #28 {AND #23-#25} AND {OR #26-#27} |
| Scopus | (((TITLE-ABS-KEY(“multi-drug resistant tuberculosis“)) OR (TITLE-ABS-KEY(“MDR-TB“)) OR (TITLE-ABS-KEY(“multidrug resistant tuberculosis“)) OR (TITLE-ABS-KEY(“drug-resistant tuberculosis“)) OR (TITLE-ABS-KEY(“XDR-TB“)) OR (TITLE-ABS-KEY(“RR-TB“)) OR (TITLE-ABS-KEY(“DR-TB“)) OR (TITLE-ABS-KEY(“extensively drug resistant tuberculosis“))) AND ((TITLE-ABS-KEY("adverse*")) OR (TITLE-ABS-KEY(“side effect”)) OR (TITLE-ABS-KEY(“safety”)) OR (TITLE-ABS-KEY("adverse drug reaction")) OR (TITLE-ABS-KEY("tolerability")) OR (TITLE-ABS-KEY("toxicity"))) AND ((TITLE-ABS-KEY("second-line anti-tuberculosis")) OR (TITLE-ABS-KEY("levofloxacin")) OR (TITLE-ABS-KEY("moxifloxacin")) OR (TITLE-ABS-KEY("bedaquiline")) OR (TITLE-ABS-KEY("linezolid")) OR (TITLE-ABS-KEY("clofazimine")) OR (TITLE-ABS-KEY("cycloserine")) OR (TITLE-ABS-KEY("terizidone")) OR (TITLE-ABS-KEY("ethambutol")) OR (TITLE-ABS-KEY("pyrazinamide")) OR (TITLE-ABS-KEY("delamanid")) OR (TITLE-ABS-KEY("meropenem")) OR (TITLE-ABS-KEY("imipenem")) OR (TITLE-ABS-KEY("streptomycin")) OR (TITLE-ABS-KEY("amikacin")) OR (TITLE-ABS-KEY("ethionamide")) OR (TITLE-ABS-KEY("prothionamide")) OR (TITLE-ABS-KEY("para-aminosalicylic acid")) OR (TITLE-ABS-KEY("kanamycin")) OR (TITLE-ABS-KEY("capreomycin")) OR (TITLE-ABS-KEY("amoxicillin potassium clavulanate combination")) OR (TITLE-ABS-KEY("thioacetazone")) OR (TITLE-ABS-KEY("clarithromycin")) OR (TITLE-ABS-KEY("high-dose isoniazid")) OR (TITLE-ABS-KEY("imipenem/cilastatin"))) AND (TITLE-ABS-KEY("human immunodeficiency virus")) OR (TITLE-ABS-KEY("HIV")) OR (TITLE-ABS-KEY("acquired immunodeficiency syndrome")) OR (TITLE-ABS-KEY("immunosuppress*"))) |
| ProQuest | (("multi-drug resistant tuberculosis" OR "MDR-TB" OR "multidrug resistant tuberculosis" OR "drug resistant tuberculosis" OR “DR-TB” OR “XDR-TB” OR “RR-TB” OR (MESH(tuberculosis, multidrug resistant))) AND ("adverse*" OR “side effect” OR “safety” OR "adverse drug reaction" OR “adverse events” OR "tolerability" OR "toxicity" OR (MESH("adverse drug reaction reporting systems" OR “safety” OR “chemically induced disorders” OR “poisoning”))) AND ((MESH(“human immunodeficiency virus proteins" OR "acquired immunodeficiency syndrome" OR "hiv")) OR "human immunodeficiency virus" OR "HIV" OR "acquired immunodeficiency syndrome" OR "immunosuppress*") AND ("second-line anti-tuberculosis" OR "levofloxacin" OR "moxifloxacin" OR "bedaquiline" OR "linezolid" OR "clofazimine" OR "cycloserine" OR "terizidone" OR "ethambutol" OR "pyrazinamide" OR "delamanid" OR "meropenem" OR "imipenem" OR "streptomycin" OR "amikacin" OR "ethionamide" OR "prothionamide" OR "para-aminosalicylic acid" OR "kanamycin" OR "capreomycin" OR "amoxicillin potassium clavulanate combination" OR "thioacetazone" OR "clarithromycin" OR "high-dose isoniazid" OR "imipenem/cilastatin" OR (MESH("levofloxacin" OR "moxifloxacin" OR "bedaquiline" OR "linezolid" OR "clofazimine" OR "cycloserine" OR "terizidone" OR "ethambutol" OR "pyrazinamide" OR "delamanid" OR "meropenem" OR "imipenem" OR "streptomycin" OR "amikacin" OR "ethionamide" OR "prothionamide" OR "para-aminosalicylic acid" OR "kanamycin" OR "capreomycin" OR "amoxicillin potassium clavulanate combination" OR "thioacetazone" OR "clarithromycin")))) |
| Google Scholar | (((("multi-drug-resistant" OR "drug-resistant”) AND (“tuberculosis”)) OR “DR-TB” OR “MDR-TB” OR “RR-TB” OR “XDR-TB”) AND ("human immunodeficiency virus" OR "acquired immunodeficiency syndrome" OR "immunosuppress*" OR "HIV" OR "AIDS")) |
| MEDLINE, CINAHL | S1 ((MH "Drug-Related Side Effects and Adverse Reactions+") OR (MH "Adverse Drug Reaction Reporting Systems") OR (MH "Chemically-Induced Disorders+") OR (MH "Safety+") OR (MH "Drug Tolerance+") OR (MH "Poisoning+") OR ("adverse events" OR "adverse effects" OR "adverse drug reaction" OR "side effect"))  S2 ((MH "Human Immunodeficiency Virus Proteins+") OR (MH "HIV+") OR (MH "Acquired Immunodeficiency Syndrome") OR (MH "HIV Infections+"))  S3 ((MH "Fluoroquinolones+") OR (MH "Moxifloxacin") OR (MH "Ofloxacin+") OR (MH "Linezolid") OR (MH "Clofazimine") OR (MH "Cycloserine") OR (MH "Ethambutol") OR (MH "Pyrazinamide") OR (MH "Carbapenems+") OR (MH "Kanamycin+") OR (MH "Amikacin") OR (MH "Streptomycin+") OR (MH "Isonicotinic Acids+") OR (MH "Capreomycin") OR (MH "Thioacetazone") OR (MH "Amoxicillin-Potassium Clavulanate Combination") OR (MH "Clarithromycin") OR (MH "Cilastatin, Imipenem Drug Combination")) OR (("second-line anti-tuberculosis" OR "bedaquiline" OR "terizidone" OR "delamanid" OR "para-aminosalicylic acid" OR "high-dose isoniazid"))  S4 ((MH "Extensively Drug-Resistant Tuberculosis") OR (MH "Tuberculosis, Multidrug-Resistant+") OR ("multi-drug resistant tuberculosis" OR "MDR-TB" OR "multidrug resistant tuberculosis" OR "drug resistant tuberculosis"))  S5 S1 AND S2 AND S3 AND S4 |

**Study eligibility criteria**

During literature search, we discovered a total of 16 studies[15–30] which required additional data to be eligible for inclusion in this systematic review. Among them, 14 studies did not stratify the adverse events (AEs) according to human immunodeficiency virus (HIV) co-infection status, while an additional two did not report the number of HIV-negative patients experiencing AEs. Upon attempted contacts, two authors[20,26] responded to the queries and provided additional data for analyses. The remaining 14 studies[15–19,21–25,27–30] were excluded from this systematic review.

**S3 Table.** PICOTS framework

| **Item** | **Definition** |
| --- | --- |
| **P**opulation | DR-TB patients receiving second-line anti-TB treatments |
| **I**ndex prognostic factor | HIV co-infection |
| **C**omparator prognostic factor | None |
| **O**utcome | Occurrence of AE, serious AEs, and specific AEs |
| **T**iming | Prognostic factor: at any time (i.e. history of HIV co-infection or newly diagnosed HIV)  Outcome: at any point during the study’s timeline |
| **S**etting | To provide prognostic information about the burden of HIV co-infection on AE occurrences following second-line anti-TB treatments in DR-TB patients. This information may help clinicians in predicting the likeliness of AEs, serious AEs, and specific types of AEs |

**AE,** adverse event; **DR-TB,** drug-resistant tuberculosis; **HIV,** human immunodeficiency virus; **PICOTS**, population, index prognostic factor, comparator, outcome, timing, and setting.

**Data extraction and quality assessment**

*Definition of drug-resistant tuberculosis (DR-TB)*

In this study, we defined drug-resistant tuberculosis (DR-TB) according to the international expert consensus proposed by Magiorakos et al.[31] DR-TB were further dichotomized into multidrug-resistant TB (MDR-TB), extensively drug-resistant TB (XDR-TB), and pandrug-resistant TB (PDR-TB).[31] MDR-TB was defined as the emergence of resistance pattern of TB bacilli to at least one first-line anti-TB drug, including rifampicin, isoniazid, pyrazinamide, ethambutol, and streptomycin[31,32]; encompassing both rifampicin-resistant TB and isoniazid-resistant TB. Meanwhile, XDR-TB was defined as resistance pattern of TB bacilli to at least one anti-TB drug in all but two or fewer drug categories, whereas PDR-TB was defined as resistance to all anti-TB agents.[31] Polydrug-resistant TB was classified on a case-by-case basis according to each study’s operational definition, where multiple drug resistance to first-line anti-TB drugs was classified into MDR-TB, while the opposite was termed as XDR-TB.

*Quality assessment*

The included studies were further assessed for methodological quality using the Risk of Bias tool in Non-randomized Studies of Intervention (ROBINS-I) tool for interventional studies, Newcastle-Ottawa Scale (NOS) for cohort studies **(S3 Table),** and the modified NOS checklist for cross-sectional studies **(S4 Table).**

**S4 Table.** Signaling questions for methodological quality assessments of cohort studies using the Newcastle-Ottawa Scale

| Item No. | Quality assessment criteria | Statement | Score (Total 9) |
| --- | --- | --- | --- |
| Selection | | | |
| 1 | Representativeness of the exposed cohort | Random or consecutive recruitments of participants or population-based study | One star |
|  |  | Selected group of users | Zero star |
|  |  | No description | Zero star |
| 2 | Selection of the non-exposed cohort | Drawn from the same community as the exposed cohort | One star |
|  |  | Drawn from different community | Zero star |
|  |  | No description | Zero star |
| 3 | Ascertainment of exposure | Medical records and/or structured interview | One star |
|  |  | Self-report | Zero star |
|  |  | No description | Zero star |
| 4 | Demonstration that outcome of interest was not present at start of study | Yes | One star |
|  |  | No | Zero star |
| Comparability | | | |
| 5 | Study controls for age/sex | Yes | One star |
|  |  | No | Zero star |
| 6 | Study controls for at least 3 additional factors:   - ART usage - Baseline CD4 count - BMI - Education - Employment status - Marital status - Previous TB treatment or outcome - Race - Resistance pattern - Weight | Yes | One star |
|  |  | No | Zero star |
| Outcome | | | |
| 7 | Assessment of outcome | Standardized assessment or confirmation of adverse events (not self-reported) | One star |
|  |  | Self-report | Zero star |
|  |  | No description | Zero star |
| 8 | Was follow-up long enough for outcomes to occur? | Yes | One star |
|  |  | No | Zero star |
| 9 | Adequacy of follow up of cohorts | Complete follow up, or subjects lost to follow up unlikely to introduce bias - small number lost $\boldsymbol{\leq}$20% or description of those lost suggesting no different from those followed-up | One star |
|  |  | Loss of follow up >20% subjects or potential bias arising from follow-up loss | Zero star |
|  |  | No description | Zero star |

**S5 Table.** Signaling questions for methodological quality assessments of cross-sectional studies using the modified Newcastle-Ottawa Scale.

| Item No. | Quality assessment criteria | Statement | Score (Total 10) |
| --- | --- | --- | --- |
| Selection | | | |
| 1 | Representativeness of the sample | All eligible subjects were included, or random sampling was utilized to recruit participants | One star |
|  |  | Selected group of users | Zero star |
|  |  | No description | Zero star |
| 2 | Sample size | Justified and satisfactory | One star |
|  |  | Not justified | Zero star |
| 3 | Non-respondents | Comparability between respondents and non-respondents’ characteristics is established, and the response rate is satisfactory | One star |
|  |  | The response rate is unsatisfactory, or the comparability between respondents and non-respondents is unsatisfactory. | Zero star |
|  |  | No description of the response rate or the characteristics of the responders and the non-responders | Zero star |
| 4 | Ascertainment of exposure (risk factors) | Validated measurement tool | Two stars |
|  |  | Non-validated measurement tool and the tool is available/described | One star |
|  |  | No description | Zero star |
|  |  | No | Zero star |
| Comparability | | | |
| 5 | Study controls for age/sex | Yes | One star |
|  |  | No | Zero star |
| 6 | Study controls for at least 3 additional factors:   - ART usage - Baseline CD4 count - BMI - Education - Employment status - Marital status - Previous TB treatment or outcome - Race - Resistance pattern - Weight | Yes | One star |
|  |  | No | Zero star |
| Outcome | | | |
| 7 | Assessment of outcome | Independent blind assessment | Two stars |
|  |  | Standardized assessment or confirmation of adverse events (not self-reported) | Two stars |
|  |  | Self-report | One star |
|  |  | No description | Zero star |
|  |  | No description | Zero star |
| 8 | Statistical test | The statistical test used to analyse the data is clearly described and appropriate, and the measurement of the association is presented, including confidence intervals and the probability level (p value) | One star |
|  |  | The statistical test is not appropriate, not described or incomplete | Zero star |

**Statistical analysis**

In this review, we approximated the reported adjusted odds ratio (OR) and adjusted hazard ratio (HR) to relative risks (RRs). For studies reporting adjusted OR as their effect measure, ORs were approximated to relative risks (RRs) using the formula provided by the Cochrane Handbook ver. 6.0[33] as follows: $RR= \frac{OR}{1-ACR x (1-OR)}$, where ACR is the baseline risk. On the other hand, HRs were converted to RRs by using the formula provided by VanderWeele et al[1] as follows: $RR= \frac{1-{0.5}^{\sqrt{HR}}}{1-{0.5}^{\sqrt{\frac{1}{HR}}}}$. These approaches have been proven to yield substantially lower bias ratio than interchangeable use between effect measures. For HR-to-RR approximation, when the outcome probability of both exposed (p_1_) and non-exposed (p_0_) cohorts falls between 0.2-0.8, the transformation bias is approximated to be at most 16% (i.e. a factor of 1.16).[1] This transformation bias may escalate up to 45% (i.e. a factor of 1.45) for outcome probability between 0.1-0.9[1] and up to 93% (i.e. a factor of 1.93) for outcome probability between 0.05-0.95. Although the anticipated bias may be quite concerning for outcome probability between 0.05-0.95, this approach still proved to be worthwhile considering that the untransformed HR may be biased for the RR by a factor of 1.80, 2.47, and 19.00 for outcome probabilities between 0.2-0.8, 0.1-0.9, and 0.05-0.95, respectively.[1] Furthermore, only one study[34] reported outcome probability between 0.9-0.95, thus further minimizing the potential bias arising from effect sizes conversion. Additionally, we performed subgroup analysis based on reported effect size to explore the impact of these data approximations, which result was elucidated in detail in the ‘Results’.

**RESULTS**

**S6 Table.** Characteristics of the included studies^a^

| **No** | **Author; Year** | **Recruitment period** | **Study characteristics** | | | | **Subject characteristics** | | | | | **Duration of follow up (months)** | **RoB score^b^** |
| --- | --- | --- | --- | --- | --- | --- | --- | --- | --- | --- | --- | --- | --- |
|  |  |  | **Study design** | **Location** | **Exposures (n)** | **Sample size (n)** | | **Age (years)** | **Male; n (%)** | **DR-TB pattern** | **HIV+; n (%) [ART; n (%)]** |  |  |
| **Interventional studies** | | | | | | | | | | | | | |
| 1 | Conradie F; 2020[35] | 16 Apr 2015 – 15 Nov 2017 | NRSI | South Africa | **TB:** BDQ (109), PTO (109), LZD (109)  **HIV:** 3TC (27), ABC (17), ATV/r (1), AZT (1), d4T (2), EFV (1), FTC (18), LPV/r (25), NVP (17), TDF (24) | 109 | | 35 (Range 17-60) | 57 (52.3) | MDR-TB (38), XDR-TB (71) | 56 (51.4) [56 (100)] | 50 | Serious |
| **Observational studies** | | | | | | | | | | | | | |
| 2 | Smith JP; 2020[34] | 2011 - 2015 | Prospective | South Africa | **TB:** EMB (206), ETO (206), KAN (206), PZA (206), TRD (206)  **HIV:** 3TC (150), d4T (150), EFV (150), TDF | 206 | | 33 (26-41) | 75 (36.4) | MDR-TB (206) | 150 (72.8) [121 (80.7)] | 33 (26-41) | 7 |
| 3 | Letswee G; 2019[36] | 1 Mar 2017 - 1 Sept 2017 | Retrospective | South Africa | **TB:** LZD-containing regimens (27)  **HIV:** AZT (3) | 27 | | 36.0 ± 9.0 | 16 (59.3) | MDR-TB (13), XDR-TB (14) | 15 (55.6) [3 (30)] | $\leq$12 | 4 |
| 4 | Hughes J; 2019[37] | 2 Nov 2015 - 1 June 2017 | Prospective | South Africa | **TB:** BDQ (14^d^), CLO (17^d^), DLM (58^d^), EMB (35^d^), ETO (10^d^), INH (31^d^), KAN (3^d^), LVX (50^d^), LZD (23^d^), MFX (4^d^), PZA (54^d^), TRD (51^d^)  **HIV:** NR | 58 | | 35 (28-42) | 32 (36.4) | MDR-TB (58) | 46 (79) [38 (83)] | $\leq$6 | 7 |
| 5 | Merid MW et al; 2019[38] | Sep 2010 - Dec 2017 | Retrospective | Ethiopia | **TB:** CAP (570), CS (570), EMB (105), ETO (170), LVX (570), PTO (400), PZA (570)  **HIV:** NR | 570 | | 28 (23-38) | 324 (56.8) | DR-TB | 154 (27.0) [148 (96.7)] | 8.23 (2.66-23.33) | 9 |
| 6 | Olayanju O; 2019[39] | Apr 2014 - Apr 2018 | Prospective | South Africa | **TB:** BDQ (63), CLO (62), DLM (8), EMB (19), ETO (14), INH (20), LVX (61), LZD (63), MFX (10), PAS (60), PZA (61), TRD (60)  **HIV:** NR | 63 | | 37 (30-44) | 39 (61.9) | XDR-TB (63) | 37 (58.7) [37 (100)] | $\leq$18 | 9 |
| 7 | Sineke T; 2019[40] | 1 Feb 2015 – 1 Jan 2018 | Cross sectional | South Africa | **TB:** BDQ (42), CLO (24), EMB (24), ETO (125), INH (20), KAN (107), MFX (149), PZA (149), TRD (125)  **HIV:** EFV (94), FTC (94), TDF (94) | 149 | | 36 (29-43) | 82 (55.0) | MDR-TB (149) | 116 (77.9) [94 (81)] | NA | 9 |
| 8 | Brust JCM; 2018[41] | May 2011 - Dec 2013 | Prospective | South Africa | **TB:** EMB (206), ETO (206), KAN (206), MFX (206), PZA (206), TRD (206)  **HIV:** 3TC (121), d4T (121), EFV (121), TDF (121) | 206 | | 33 (26-41) | 75 (36.4) | MDR-TB (206) | 150 (57.7) [121 (81)] | 32 (22-37) | 8 |
| 9 | Hong H; 2018[42] | Nov 2014 - Jun 2017 | Prospective | South Africa | **TB:** AMK (89), ETO, KAN (849), MFX, PZA, TRD  **HIV:** NR | 936 | | 36.15 ± 11.04 | 505 (54.0) | MDR-TB (936) | 697 (74.5) [432 (62)] | $\leq$36 | 8 |
| 10 | Perumal R; 2018[43] | 2011 - 2013 | Retrospective | South Africa | **TB:** CAP or KAN-based regimens (215)  **HIV:** 3TC (10), ABC (2), AZT (3), d4T (5), EFV (10), TDF (163) | 215 | | 33 (28-40) | 111 (51.6) | DR-TB | 173 (80.47) [173 (100)] | KAN+TDF: 6.6 (5.6-7.8)  KAN: 6.4 (5.5-7.3)  KAN+Others: 5.3 (4.7-5.7) | 9 |
| 11 | Dela AI et al; 2017[44] | 2010 - 2013 | Retrospective | India | **TB:** CS (125), EMB (125), ETO (125), KAN (125), LVX (125), PAS (125), PZA (125)  **HIV:** NR | 125 | | 35.69 ± 12.88 | 89 (71.2) | MDR-TB (125) | 7 (5.6) [NR] | NR | 5 |
| 12 | Huerga H; 2017[45] | May 2006 - May 2012 | Retrospective | Kenya | **TB:** CAP (169), CS (169), EMB (71), KAN (169), LVX (98), OFX (71), PAS (98), PTO(169), PZA (71)  **HIV:** NR | 169 | | 29 (23-36) | 93 (55.0) | MDR-TB (169) | 43 (25.4) [21 (48.8)] | ≥24 | 8 |
| 13 | Sagwa EL; 2017[46] | 1 Jan - 31 Dec 2014 | Retrospective | Namibia | **TB:** CS (135), EMB (135), ETO (135), KAN (135), LVX (135), PZA (135)  **HIV:** 3TC, FV, NVP, TDF (44) | 135 | | 34.8 ± 12.9 | 85 (64.4) | MDR-TB (135) | 67 (49.6) [67 (100)] | $\leq$8 | 7 |
| 14 | Kelly AM; 2016[47] | May - July 2014 | Retrospective | South Africa | **TB:** EMB (121), ETO (121), KAN (121), MFX (121), PZA (121), TRD (121)  **HIV:** EFV (90), FTC (90), TDF (90) | 121 | | 33 (Range 17-63) | 59 (48.8) | MDR-TB (121) | 90 (75) [90 (100)] | NR | 5 |
| 15 | Mehta S; 2016[48] | Jan 2013 – Apr 2016 | Retrospective | India | **TB:** LZD-containing regimens (86), STM (10)  **HIV:** NR | 86 | | 25 (Range 20-35) | 41 (47.7) | MDR-TB (43), XDR-TB (43) | 17 (19.8) [NR] | NR | 5 |
| 16 | Schnippel K; 2016[49] | May 2012 - Dec 2014 | Retrospective | South Africa | **TB:** ETO (578), KAN (578), MFX (578), PZA (578), TRD (578)  **HIV:** 3TC (265), AZT (80), d4T (80), EFV (105), FTC (182), LPV/r (49), NVP, TDF (185) | 578 | | 35 (29-42) | 283 (49.0) | MDR-TB (571), XDR-TB (7) | 477 (82.5) [209 (43.8)] | $\leq$6 | 9 |
| 17 | Sogebi AO; 2016[50] | 1 Jan 2015 - 31 Dec 2015 | Prospective | Nigeria | **TB:** CS (70), KAN (70), LVX (70), PTO (70), PZA (70)  **HIV:** NR | 70 | | 34.6 ± 13.2 | 44 (62.9) | MDR-TB (70) | 9 (12.9) [9 (12.9)] | $\leq$4 | 9 |
| 18 | Avong YK; 2015[51] | 1 Feb 2012 - 31 Dec 2013 | Retrospective | Nigeria | **TB:** AMK (482), CS (482), KAN (482), LVX (482), PN (482), PTO (482), PZA (482)  **HIV:** NR | 460 | | 33 (28-42) | 285 (62.0) | MDR-TB (460) | 47 (10.2) [NR] | ≥8 | 7 |
| 19 | Meressa D; 2015[52] | Feb 2009 – Dec 2014 | Retrospective | Ethiopia | **TB:** AMK, CAP, CS, ETO, KAN, LVX, PAS, PZA (612)  **HIV:** 3TC (112), AZT (41), d4T (28), EFV (95), TDF (46) | 612 | | 27 (22-36) | 325 (53.1) | MDR-TB (612) | 133 (21.7) [115 (86.5)] | $\geq$24 | 9 |
| 20 | Modongo C; 2015[53] | 1 Jan 2011 - 30 Dec 2012 | Retrospective | Botswana | **TB:** AMK (28), CS (28), ETO (28), LVX (28), PZA (28)  **HIV:** 3TC, AZT, EFV, FTC, NVP, TDF | 28 | | 44 ± 18 | 16 (57.1) | MDR-TB (28) | 16 (57.1) [16 (100)] | NR | 4 |
| 21 | Sagwa EL; 2015[54] | Jun 2004 - Mar 2014 | Retrospective | Namibia | **TB:** AMC (9), AMK (51), CAP (16), CLO (8), CLR (3), CPFX (48), CS (278), EMB (161), ETO (327), INH (11), KAN (281), LVX (298), OFX, PAS (18), PZA (331), R (34)  **HIV:** 3TC (109), ABC (1), AZT (33), d4T (14), EFV (87), LPV/r (4), NVP (19), TDF 63) | 353 | | 36.4 ± 11.3 | 198 (56.1) | MDR-TB (353) | 164 (46.5) [132 (87.2)] | NR | 8 |
| 22 | Sagwa EL; 2012-2014[20,55–57] | Jan 2008 - Feb 2010 | Retrospective | Namibia | **TB:** AMC (1), AMK (21), CAP (4), CLO (1), CPFX (19), CS (29), EMB (36), ETO (54), INH (4), KAN (32), LVX (39), PAS (5), PZA (55), R (13), STM (3)  **HIV:** AZT (5), d4T (6), EFV (10), LPV/r (3) | 59^c^ | | 34.7 ± 9.4 | 38 (64) | MDR-TB (54), XDR-TB (1) | 31 (52.5) [13 (41.9)] | NR | 8 |
| 23 | Charles M; 2014[58] | 3 Mar 2010 - 28 Mar 2013 | Prospective | Haiti | **TB:** AMC (2), CAP (14), CS (108), ETO, INH (44), KAN (96), LVX (86), MFX (14), PAS (64), PZA (106)  **HIV:** LPV/r-based regimens (15) | 110 | | 28 (23-37) | 50 (45.5) | MDR-TB (110) | 27 (24.5) [27 (100)] | $\leq$12 | 9 |
| 24 | Conradie F; 2014[59] | Aug 2009 - Aug 2011 | Prospective | South Africa | **TB:** NR  **HIV:** NR | 246 | | Median: 37 | 17 113 (45.9) | MDR-TB (246) | 199 (80.1) [88 (35.7)] | 14.86 (10.26-20.65) | 7 |
| 25 | Modongo C; 2014[60] | 1 Jan 2006 - 30 Jun 2012 | Retrospective | Botswana | **TB:** AMK, CS, ETO, LVX, PZA  **HIV:** NR | 437 | | 38 (31-49) | 240 (54.9) | MDR-TB (437) | 288 (65.9) [267 (92.7)] | NR | 9 |
| 26 | Padayatchi N; 2014[26] | Aug 2009 - Jul 2011 | Retrospective | South Africa | **TB:** AMC (41), CAP (82), CLO (50), CLR (26), EMB (74), ETO (82), INH (43), MFX (82), OFX (10), PAS (79), PZA (83), R (2), TRD (75)  **HIV:** NR | 85 | | 34 (26-42) | 50 (35.3) | XDR-TB (85) | 73 (85.9) [64 (87.7)] | $\leq$12 | 6 |
| 27 | Brust JCM; 2013[61] | 1 Nov 2008 - 15 Apr 2011 | Retrospective | South Africa | **TB:** CS (91), EMB (91), ETO (91), KAN (91), OFX (91), PZA (91)  **HIV:** 3TC (74), AZT, d4T, EFV (74), TDF | 91 | | 34 (29-41) | 36 (39.6) | MDR-TB (91) | 76 (83.5) [74 (97.4)] | 21.44 (15.65-23.93) | 6 |
| 28 | Seddon JA; 2013[62] | Jan 2009 – Dec 2010 | Retrospective | South Africa | **TB:** AMK (82), CAP (9), STM (1)  **HIV:** NR | 93 | | 43 (20-110) months | 45 (47.9) | MDR-TB (93) | 28 (30.1) [20 (71.4)] | NR | 8 |
| 29 | Shean K; 2013[63] | Aug 2002 - Feb 2008 | Retrospective | South Africa | **TB:** AMC (65), AMK (3), AZM (11), CAP (104), CLO (28), CLR (77), CS (101), DDS (36), EMB (46), ETO (66), INH (41), INN (2), KAN (4), MFX (2), OFX (29), PAS (101)PZA (80), RFB (1), TRD (101)  **HIV:** 3TC (29), AZT (5), d4T (25), EFV (25), LPV/r (1), TDF (4) | 115 | | NR | 62 (53.9) | XDR-TB (115) | 48 (41.7) [34 (70.8)] | 7.3 (3.1-12.6) | 9 |
| 30 | van der Walt M; 2013[64] | 2000-2004 | Prospective | South Africa | **TB:** AMK, CPFX, CS, EMB, ETO (1390), KAN, OFX, PZA (1390), TRD  **HIV:** NA | 1390 | | 36.36 | 841 (60.5) | MDR-TB (1390) | 544 (39.1) [0 (0)] | ≥16 | 5 |
| 31 | Harris T; 2012[65] | NR | Prospective | South Africa | **TB:** CAP (1), KAN (145), STM (5)  **HIV:** NR | 151 | | 36 (Range: 14-70) | 51 (33.8) | MDR-TB (151) | 86 (57.0) [86 (100)] | $\leq$3 | 8 |
| 32 | Jacobs T; 2012[66] | Jul 2011 - Aug 2011 | Retrospective | South Africa | **TB:** AMK (350), EMB (345), ETO (350), KAN (350), OFX (350), PZA (350), TRD (337)  **HIV:** d4T, EFV, LPV/r, NVP, TDF | 350 | | 35.65 ± 11.24 | 168 (48.0) | MDR-TB (350) | 254 (72.5) [216 (85.0)] | ≥6 | 8 |
| 33 | Kvasnovsky CL; 2011[67] | Oct 2006 - Jan 2008 | Retrospective | South Africa | **TB:** CAP (4^d^), EMB (192^d^), ETO (146^d^), PAS (173^d^), PZA (199^d^), R (4^d^), TRD (93^d^)  **HIV:** NR | 206 | | 36.2 ± 11.6 | 101 (49.0) | XDR-TB (206) | 108 (52.4) [59 (54.6)] | $\leq$12 | 9 |
| 34 | Burgos M; 2005[68] | 1 Jan 1982 - 31 Dec 2000 | Retrospective | USA | **TB:** AMC (7), AMK (3), CAP (3), CLO (2), CPFX (8), CS (29), EMB (25), ETO (17), IPM (5), KAN (26), LVX (7), OFX (22), PAS (3), PZA (37), R (1), STM (28)  **HIV:** NR | 48 | | 47.2 ± 14.8 | 32 (66.7) | MDR-TB (48) | 11 (22.9) [2 (18.2)] | **HIV+:** Median 12  **HIV-:** Median 24 (Range 9-57) | 9 |

^a^Unless otherwise specified, all data are presented in n(%), mean ± standard deviation, or median (interquartile range). ^b^Risk of bias assessment of interventional studies were assessed with the Risk of Bias in Non-randomized Studies of Intervention (ROBINS-I) tool, while those of observational studies were assessed with the Newcastle-Ottawa Scale (NOS). ^c^Unpublished data, provided by request; ^d^Data were digitized with GetData Graph Digitizer ver. 2.26 (www.getdata-graph-digitizer.com).

**3TC,** lamivudine; **ABC**, abacavir; **AMC**, amoxicillin/clavulanic acid; **AMK**, amikacin; **AZM**, azithromycin; **ATV/r,** atazanavir/ritonavir; **AZT**, zidovudine; **BDQ**, bedaquiline; **CAP**, capreomycin; **CLR**, clarithromycin; **CPFX**, ciprofloxacin; **CS**, cycloserine; **d4T**, stavudine; **DDS**, dapsone; **DR-TB**, drug-resistant tuberculosis; **EFV**, efavirenz; **EMB**, ethambutol; **ETO**, ethionamide; **FOX**, cefoxitin; **FTC,** emtricitabine**; INH**, isoniazid; **INN**, thioacetazone; **IPM**, imipenem**; KAN**, kanamycin; **LPV/r**, lopinavir/ritonavir; **LVX**, levofloxacin; **LZD**, linezolid; **MDR-TB**, multidrug-resistant tuberculosis; **MFX**, moxifloxacin; **NA**, not available; **NR**, not reported; **NRSI,** non-randomized studies of intervention; **NVP**, nevirapine; **OFX**, ofloxacin; **PAS**, para-aminosalicylic acid; **PN**, pyridoxine; **PZA**, pyrazinamide; **R**, rifampicin; **RFB**, rifabutin; **STM**, streptomycin; **TDF**, tenofovir; **TRD**, terizidone; **USA**, United Stated of America; **XDR-TB**, extensively drug-resistant tuberculosis

**S7 Table**. Results of quality assessment of included cohort studies.

| **Category** | **Criteria** | Smith JP, 2019 | Letswee G, 2019 | Hughes J, 2019 | Merid MW, 2019 | Olayanju O, 2019 | Brust JCM, 2018 | Hong H, 2018 | Perumal R, 2018 | Dela AI, 2017 | Huerga H, 2017 | Sagwa EL; 2017 | Kelly AM; 2016 | Mehta S; 2016 | Schnippel K; 2016 | Sogebi AO; 2016 | Avong YK; 2015 |
| --- | --- | --- | --- | --- | --- | --- | --- | --- | --- | --- | --- | --- | --- | --- | --- | --- | --- |
| **Selection** | Representativeness of the exposed cohort | * | - | * | * | * | * | * | * | * | * | * | * | * | * | * | * |
|  | Selection of the non-exposed cohort | * | * | * | * | * | * | * | * | * | * | * | * | * | * | * | * |
|  | Ascertainment of exposure | - | * | * | * | * | * | * | * | - | * | * | * | * | * | * | * |
|  | Demonstration that outcome of interest was not present at start of study | - | - | - | * | * | * | - | * | - | - | * | - | - | * | * | - |
| **Comparability** | Study controls for age/sex | * | * | * | * | * | * | * | * | * | * | * | * | * | * | * | * |
|  | Study controls for at least 3 additional factors | * | - | * | * | * | * | * | * | - | * | - | - | - | * | * | - |
| **Outcome** | Assessment of outcome | * | * | * | * | * | - | * | * | - | * | * | * | * | * | * | * |
|  | Was follow-up long enough for outcomes to occur? | * | - | - | * | * | * | * | * | * | * | - | - | - | * | * | * |
|  | Adequacy of follow up of cohorts | * | - | * | * | * | * | * | * | * | * | * | - | - | * | * | * |
| **Total** | | 7 | 4 | 7 | 9 | 9 | 8 | 8 | 9 | 5 | 8 | 7 | 5 | 5 | 9 | 9 | 7 |

**S7 Table.** Results of quality assessment of included cohort studies (cont.)

| **Category** | **Criteria** | Meressa D; 2015 | Modongo C, 2015 | Sagwa EL; 2015 | Sagwa EL; 2012-2014 | Charles M; 2014 | Conradie F; 2014 | Modongo C; 2014 | Padayatchi N; 2014 | Brust JCM, 2013 | Seddon JA, 2013 | Shean K; 2013 | Van der Walt, 2013 | Harris T; 2012 | Jacobs T; 2012 | Kvasnovsky CL; 2011 | Burgos M; 2005 |
| --- | --- | --- | --- | --- | --- | --- | --- | --- | --- | --- | --- | --- | --- | --- | --- | --- | --- |
| **Selection** | Representativeness of the exposed cohort | * | - | * | * | * | * | * | * | * | * | * | * | * | * | * | * |
|  | Selection of the non-exposed cohort | * | - | * | * | * | * | * | * | * | * | * | * | * | * | * | * |
|  | Ascertainment of exposure | * | - | * | * | * | - | * | * | * | * | * | - | * | * | * | * |
|  | Demonstration that outcome of interest was not present at start of study | * | - | * | - | * | * | * | - | - | - | * | - | * | - | * | * |
| **Comparability** | Study controls for age/sex | * | * | * | * | * | * | * | * | * | * | * | * | * | * | * | * |
|  | Study controls for at least 3 additional factors | * | - | - | * | * | * | * | - | - | * | * | - | - | * | * | * |
| **Outcome** | Assessment of outcome | * | * | * | * | * | - | * | * | * | * | * | - | * | * | * | * |
|  | Was follow-up long enough for outcomes to occur? | * | * | * | * | * | * | * | * | * | * | * | * | * | * | * | * |
|  | Adequacy of follow up of cohorts | * | * | * | * | * | * | * | - | - | * | * | * | * | * | * | * |
| **Total** | | 9 | 4 | 8 | 8 | 9 | 7 | 9 | 6 | 6 | 8 | 9 | 5 | 8 | 8 | 9 | 9 |

**S8 Table.** Results of quality assessment of included cross-sectional studies.

| **Category** | **Criteria** | Sineke T, 2019 |
| --- | --- | --- |
| **Selection** | Representativeness of the sample | * |
|  | Sample size | * |
|  | Non-respondents | - |
|  | Ascertainment of exposure (risk factor) | ** |
| **Comparability** | Study controls for age/sex | * |
|  | Study controls for at least 3 additional factors | * |
| **Outcome** | Assessment of outcome | ** |
|  | Statistical test | * |
| **Total** | | 9 |

**S1 Fig.** Results of risk of bias assessment of included non-randomized studies of intervention.

**
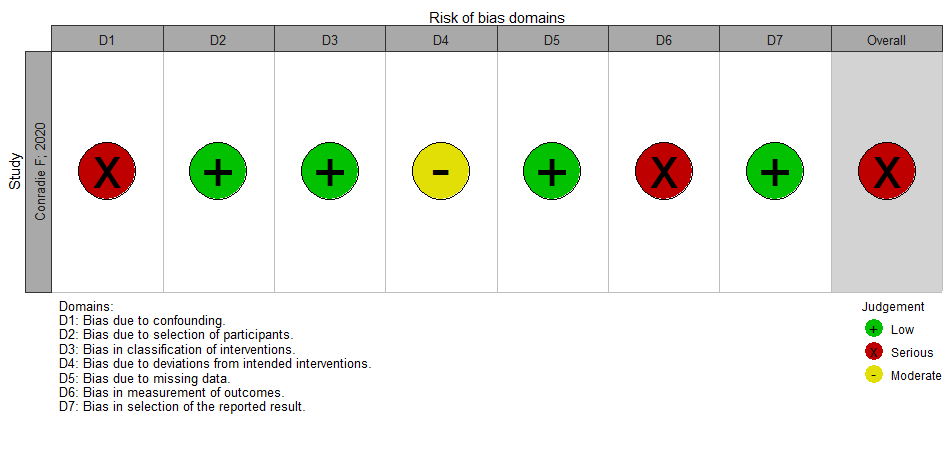
**

**S9 Table.** Study-specific outcomes on the risk of developing adverse events.

| **Author; Year** | **Adjusted variables** | **Risks of developing AEs**  **(RR [95% CI])^a^** | | **Notes** |
| --- | --- | --- | --- | --- |
|  |  | **Unadjusted^b^** | **Adjusted** |  |
| **Interventional studies** | | | | |
| Conradie F; 2020[35] |  | 1.00 (1.00-1.00) |  | Adjustment of outcome is not necessary as  Serious AE: RR 0.85 (95% CI: 0.38-1.93)  Severe AE: RR 1.22 (95% CI: 0.88-1.71) |
| **Observational studies** | | | | |
| Smith JP; 2020[34] | Age, Sex, Weight, Alcohol, Smoking, History of mining, Healthcare worker, History of incarceration, Baseline viral load | 0.98 (0.91-1.06) | 1.00 (0.82-1.21)^c^ | Potential overlapping populations with Brust et al[41,61], Hong et al[42], Letswee et al[36], and Perumal et al[43] |
| Letswee G; 2019[36] |  | 12.19 (0.77-194.03) |  | Potential overlapping population with Hong H; 2018[42]. Only hematologic AEs were reported. |
| Hughes J; 2019[37] |  | 1.19 (0.84-1.67) |  |  |
| Merid MW et al; 2019[38] | Age, Sex, Comorbidities, Functional status, TB complications, Education status, Hemoglobin level | 0.89 (0.75-1.06) | 0.98 (0.58-1.66)^c^ | Only serious AEs were reported. |
| Olayanju O; 2019[39] | Age, Sex, Weight, Days hospitalized, Time to positivity, Duration of LZD, PZA use, number of TB drugs | 1.01 (0.84-1.21) | 1.61 (0.92-2.87)^c^ |  |
| Sineke T; 2019[40] |  | OR 1.36 (0.59-3.16) |  | CD4 count ≥50 vs <50 cells/mm^3^: OR 2.14 (95% CI: 0.88-5.20)^b^ |
| Brust JCM; 2018[41] |  |  |  | No number of patients experiencing any AEs were reported. The study only reported multiple specific AEs^g^ |
| Hong H; 2018[42] |  | 1.17 (0.97-1.41) |  | Potential overlapping population with Smith et al[34] and Letswee et al[36]. Only AEs on ototoxicity were reported |
| Perumal R; 2018[43] | Age, Sex, Baseline eGFR | 1.98 (0.97-4.02) | 1.89 (1.19-2.94)^d,f^ | Only AEs on nephrotoxicity were reported. |
| Dela AI et al; 2017[44] |  | 1.26 (0.77-2.06) |  |  |
| Huerga H; 2017[45] |  | No significant difference in total AEs and time to AEs onset by HIV status | |  |
| Sagwa EL; 2017[46] |  | HR 2.71 (1.49-4.94)^f^ | HR 2.51 (1.31-4.82)^f^ | Potential overlapping population with Sagwa EL; 2015[54]. Only AEs on nephrotoxicity were reported. |
| Kelly AM; 2016[47] |  | 1.58 (0.78-3.20) |  | No significant difference in total AEs by HIV status based on patient interviews (p=0.277) or medical records (p=0.098) |
| Mehta S; 2016[48] |  | 1.35 (0.64-2.88) |  |  |
| Schnippel K; 2016[49] |  | 1.74 (1.21-2.49)^d,f^ |  | No number of patients experiencing any AEs were reported. The study only reported multiple specific AEs^g^ |
| Sogebi AO; 2016[50] | Sex | 3.08 (1.40-6.80) | 3.66 (1.93-6.93)^d^ | Only AEs on nephrotoxicity were reported. |
| Avong YK; 2015[51] | Age, Sex, Weight, Treatment center | 1.01 (0.72-1.42) | 1.30 (0.95-1.78)^d^ |  |
| Meressa D; 2015[52] |  | No significant difference in AE rates between HIV-positive and HIV-negative patients (p=0.478) | |  |
| Modongo C; 2015[53] |  | 0.76 (0.29-2.02) |  | Potential overlapping population with Modongo C; 2014[60]. Only AEs on ototoxicity were reported. |
| Sagwa EL; 2015[54] | Age, Sex | 1.17 (0.98-1.39)^e^ | 1.15 (0.99-1.34)^d,e^ | The author has confirmed that potential overlapping population with Sagwa EL; 2012-2014[20,55–57] is negligible. Only AEs on ototoxicity were reported |
| Sagwa EL; 2012-2014[20,55–57] | Age, Sex | 1.02 (0.85-1.22)^e^ | 0.92 (0.22-1.61)^d,e^ | The author has confirmed that potential overlapping population with Sagwa EL; 2015[54] is negligible |
| Charles M; 2014[58] |  | 0.85 (0.55-1.31) |  | Only AEs on peripheral neuropathy were reported |
| Conradie F; 2014[59] | Age, Sex, Prior exposure to d4T/INH within 6 months | 1.00 (0.46-2.16) | 1.08 (0.50-2.35)^d^ |  |
| Modongo C; 2014[60] | Age, Sex, TB treatment history, Creatinine clearance, Duration and dose of AMK | 1.13 (0.96-1.33) | 1.12 (0.94-1.32)^c^ | Potential overlapping population with Modongo C; 2015[53]. Only AEs on ototoxicity were reported.  CD4 count ≥50 vs <50 cells/mm^3^: OR 1.32 (95% CI: 0.83-2.12) |
| Padayatchi N; 2014[26] |  | 1.48 (0.21-10.65) |  |  |
| Brust JCM; 2013[61] |  |  |  | No number of patients experiencing any AEs were reported. The study only reported multiple specific AEs^g^ |
| Seddon JA; 2013[62] |  | 1.50 (0.84-2.68) |  |  |
| Shean K; 2013[63] |  | 1.07 (0.78-1.45) |  |  |
| van der Walt M; 2013[64] |  | 1.45 (0.95-2.19) |  | Only serious AEs were reported. |
| Harris T; 2012[65] |  | 1.68 (1.22-2.31) |  | Only AEs on ototoxicity were reported. |
| Jacobs T; 2012[66] |  | 2.62 (1.47-4.67) |  |  |
| Kvasnovsky CL; 2011[67] |  | 0.30 (0.15-0.58) |  | Only serious AEs were reported. |
| Burgos M; 2005[68] |  | 0.72 (0.25-2.06) |  | Only serious AEs were reported. |

^a^Unless explicitly stated, all outcomes are presented in risk ratios along with their 95% CIs. ^b^Calculated from binary data[69]; ^c^Calculated using the formula provided by Cochrane Handbook ver. 6.0[33]; ^d^Calculated using the formula provided by VanderWeele et al.[1]; ^e^Unpublished data, provided by request. ^f^Outcomes were pooled using fixed-effect meta-analysis from the originally reported subset-specific effect size. ^g^To prevent model selection bias, we excluded these studies from the primary analysis and later included these studies in the secondary outcome on specific AEs.

**AE,** adverse event; **AMK,** amikacin; **CI,** confidence interval; **d4T,** stavudine; **HR,** hazard ratio; **HIV**, human immunodeficiency virus; **LZD,** linezolid; **PZA**, pyrazinamide; **RR**, risk ratio; **TB,** tuberculosis

**S2 Fig.** Pooled unadjusted effects on the association between HIV co-infection and the occurrence of adverse events. **CI**, confidence interval; **RR**, risk ratio

**
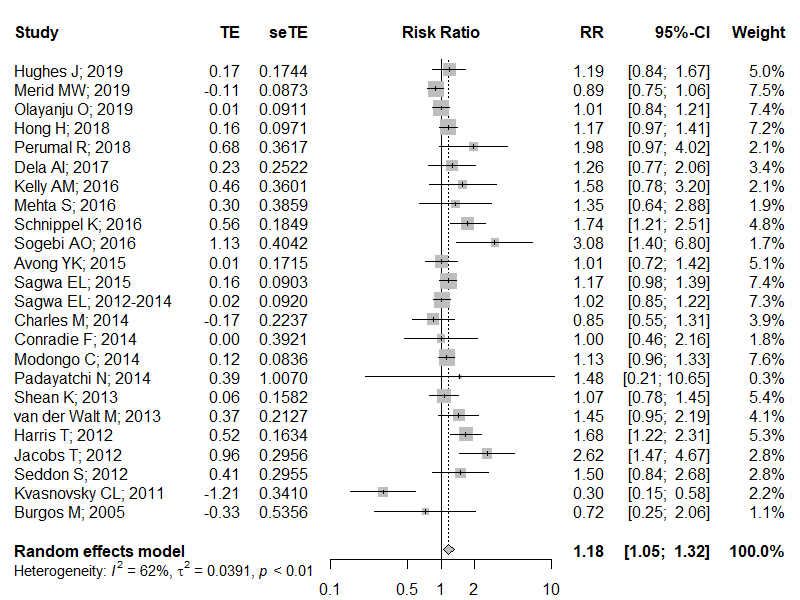
**

**S3 Fig.** Results of sensitivity analysis for the pooled adjusted effects on the association between HIV co-infection and adverse events occurrence.**CI,** confidence interval; **I2**, I-squared value; **RR,** risk ratio.

**
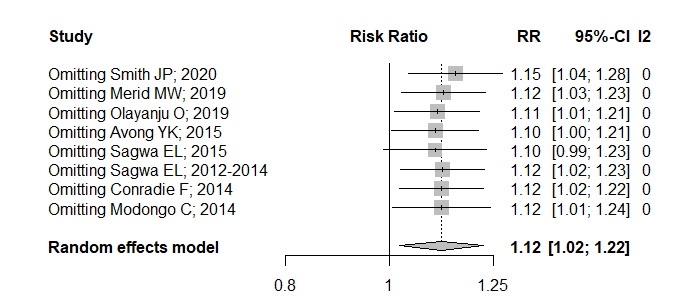
**

**S4 Fig.** Results of subgroup analyses of the pooled unadjusted effects on the association between HIV co-infection and adverse events occurrence. **AE,** adverse event; **ART,** antiretroviral therapy; **CD4,** cluster of differentiation 4; **CI,** confidence interval; **MDR-TB,** multidrug-resistant tuberculosis; **RR,** risk ratio; **TB,** tuberculosis; **XDR-TB,** extensively drug-resistant tuberculosis

**
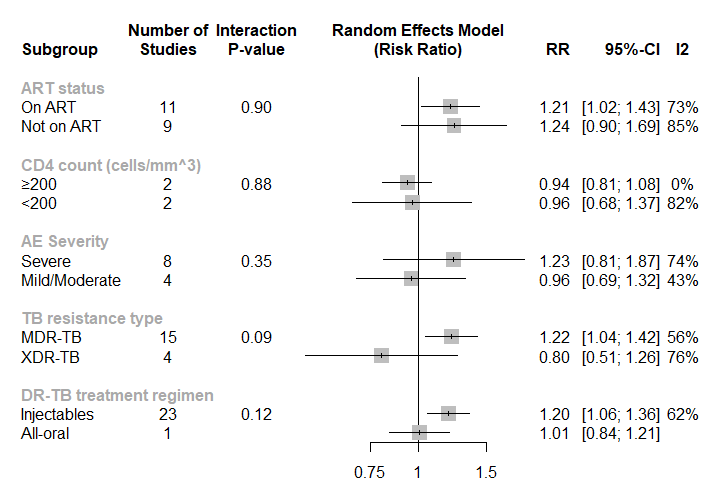
**

**S5 Fig.** Contour-enhanced funnel plot with pseudo 95% confidence interval indicating no publication bias (as shown by symmetry) for the pooled adjusted effects on the association between HIV co-infection and adverse events occurrence.


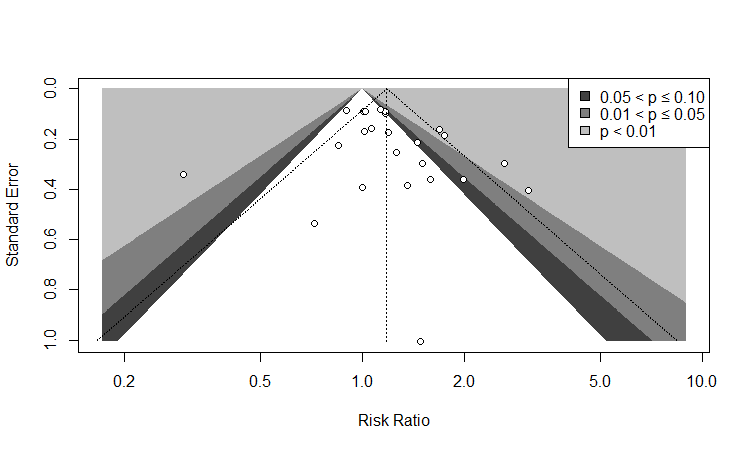


**S10 Table.** Summary of meta-analysis on specific AE outcomes^a^

| Outcome | # Cohorts | Events/N | | RR (95% CI) | I^2^ |
| --- | --- | --- | --- | --- | --- |
|  |  | HIV+ | HIV- |  |  |
| Clinical AE |  |  |  |  |  |
| Cardiovascular AE |  |  |  |  |  |
| Angina/Chest pain | 2 | 56/196 | 27/68 | 0.55 (0.18-1.66) | 69%* |
| Prolonged QTc interval | 2 | 13/94 | 4/78 | 1.03 (0.37-2.87) | 0% |
| Dermatologic AE |  |  |  |  |  |
| Dry skin | 1 | 2/73 | 0/12 | 0.88 (0.04-17.27) | NA |
| Hyperpigmentation | 1 | 69/150 | 21/56 | 1.23 (0.84-1.79) | NA |
| Hypopigmentation | 1 | 0/73 | 1/12 | 0.06 (0.03-1.36) | NA |
| Pruritus^b^ | 2 | 8/525 | 1/168 | 1.08 (0.23-15.06) | 0% |
| Rash^b,c,d,e^ | 5 | 120/962 | 22/252 | 1.35 (0.94-1.94) | 0% |
| Endocrine and metabolic AE |  |  |  |  |  |
| Gout | 1 | 1/254 | 0/87 | 1.04 (0.04-25.18) | NA |
| Gynecomastia^b^ | 2 | 0/77 | 5/141 | 0.36 (0.04-3.02) | 0% |
| Thyroid dysfunction^b,c^ | 5 | 57/956 | 22/394 | 1.14 (0.69-1.89) | 0% |
| Gastrointestinal AE |  |  |  |  |  |
| Abdominal pain^b,c,d,e^ | 4 | 70/706 | 31/250 | 1.30 (0.55-3.11) | 58%* |
| Anorexia | 1 | 6/46 | 0/12 | 3.60 (0.22-59.73) | NA |
| Constipation^d,e^ | 1 | 1/31 | 0/26 | 2.53 (0.11-59.63) | NA |
| Diarrhea^c,d,e^ | 4 | 64/275 | 23/161 | 1.47 (0.72-3.00) | 37% |
| Dyspepsia | 3 | 38/348 | 30/166 | 0.63 (0.38-1.03) | 0% |
| Hepatitis^b^ | 2 | 13/731 | 1/188 | 1.58 (0.23-10.63) | 8% |
| Mucositis/Stomatitis^b^ | 3 | 21/777 | 2/200 | 1.47 (0.46-4.72) | 0% |
| Nausea^b,d,e^ | 6 | 88/726 | 41/279 | 1.03 (0.73-1.45) | 0% |
| Vomiting^b,c,d,e^ | 6 | 104/802 | 38/320 | 1.35 (0.93-1.94) | 0% |
| Injection site reaction^b^ | 4 | 66/649 | 18/241 | 1.11 (0.72-1.72) | 0% |
| Musculoskeletal AE |  |  |  |  |  |
| Arthralgia^b,c,d,e^ | 7 | 139/1037 | 67/478 | 0.91 (0.70-1.18) | 0% |
| Arthritis | 1 | 15/46 | 2/12 | 1.96 (0.52-7.41) | NA |
| Finger cramps | 1 | 1/254 | 0/87 | 1.04 (0.04-25.18) | NA |
| Myalgia^c^ | 3 | 72/244 | 28/135 | 1.15 (0.53-2.50) | 38% |
| Tremor^b,d,e^ | 2 | 2/81 | 1/84 | 1.53 (0.19-12.09) | 0% |
| Neurologic AE |  |  |  |  |  |
| Altered mental | 1 | 1/73 | 0/12 | 0.53 (0.02-12.15) | NA |
| Cognitive/Behavioral disorder | 1 | 4/254 | 2/87 | 0.69 (0.13-3.67) | NA |
| Headache^b,d,e^ | 6 | 29/906 | 9/351 | 1.54 (0.74-3.22) | 0% |
| Neurosensory alteration | 2 | 3/302 | 0/154 | 2.83 (0.32-25.32) | 0% |
| Peripheral neuropathy^b,c,d,e^ | 8 | 244/1262 | 108/886 | 1.49 (0.98-2.28) | 56%** |
| Seizures^b^ | 4 | 15/855 | 3/261 | 1.56 (0.49-5.02) | 0% |
| Psychiatric AE |  |  |  |  |  |
| Depression^b,d,e^ | 5 | 28/230 | 6/249 | 3.53 (1.38-9.03) | 0% |
| Disembodied feeling | 1 | 1/254 | 0/87 | 1.03 (0.04-25.18) | NA |
| Insomnia^b,c^ | 4 | 87/931 | 30/302 | 1.01 (0.74-1.38) |  |
| Loss of libido | 1 | 1/254 | 0/87 | 1.03 (0.04-25.18) | NA |
| Psychiatric disorders^b,d,e,f^ | 7 | 84/961 | 40/437 | 1.20 (0.65-2.21) | 47%* |
| Psychosis^b,d,e^ | 6 | 54/934 | 25/354 | 0.74 (0.46-1.22) | 0% |
| Respiratory AE |  |  |  |  |  |
| Dyspnea/Respiratory distress | 1 | 37/150 | 11/56 | 1.26 (0.69-2.29) | NA |
| Flu-like illness | 1 | 1/254 | 0/87 | 1.04 (0.04-25.18) | NA |
| Hiccups | 1 | 0/254 | 1/87 | 0.12 (0.004-2.80) | NA |
| Sore threat | 1 | 1/48 | 1/67 | 1.30 (0.09-21.77) | NA |
| Sensory AE |  |  |  |  |  |
| Dizziness^b,d,e^ | 6 | 37/906 | 20/351 | 0.92 (0.49-1.74) | 0% |
| Fatigue/Malaise^c,d^ | 3 | 60/229 | 28/149 | 0.50 (0.10-2.57) | 49% |
| Fever^d,e^ | 1 | 0/31 | 3/26 | 0.12 (0.01-2.23) | NA |
| Hearing loss^b,c,d,e,g^ | 12 | 787/2147 | 352/1249 | 1.44 (1.18-1.75) | 60%*** |
| Mastodynia | 1 | 1/254 | 0/87 | 1.04 (0.04-25.18) | NA |
| Tinnitus^b,c,d,e^ | 4 | 86/708 | 50/241 | 0.74 (0.48-1.15) | 29% |
| Visual alterations^d,e^ | 6 | 43/529 | 26/315 | 1.49 (0.87-2.54) | 0% |
| Systemic AE |  |  |  |  |  |
| Swelling | 2 | 41/404 | 14/143 | 1.07 (0.63-1.79) | 0% |
| Unintentional weight loss^b^ | 1 | 40/477 | 4/101 | 2.12 (0.77-5.79) | NA |
| Urinary AE |  |  |  |  |  |
| Renal impairment^b^ | 5 | 53/856 | 9/396 | 2.45 (1.20-4.98) | 0% |
| Laboratory AE |  |  |  |  |  |
| Anemia | 3 | 37/135 | 3/39 | 2.39 (0.46-12.46) | 51% |
| Elevated ALP | 1 | 69/150 | 21/56 | 1.23 (0.84-1.79) | NA |
| Elevated AST/ALT^c^ | 2 | 45/194 | 11/68 | 1.47 (0.81-2.67) | 0% |
| Elevated creatinine^c^ | 1 | 61/149 | 24/56 | 1.11 (0.79-1.57) | NA |
| Elevated TSH | 1 | 28/106 | 9/46 | 1.35 (0.69-2.63) | NA |
| Hyperkalemia^c^ | 1 | 16/149 | 10/56 | 0.60 (0.29-1.25) | NA |
| Hyperuricemia | 1 | 1/254 | 0/87 | 1.04 (0.04-25.18) | NA |
| Hypoalbuminemia | 1 | 4/46 | 2/12 | 0.52 (0.11-2.52) | NA |
| Hypocalcemia | 1 | 7.46 | 1/12 | 1.83 (0.25-13.45) | NA |
| Hypokalemia^b,c^ | 3 | 97/673 | 25/224 | 1.62 (0.86-3.06) | 21% |
| Low hemoglobin | 1 | 1/15 | 0/12 | 2.44 (0.11-54.97) | NA |
| Macrocytosis | 1 | 1/15 | 0/12 | 2.44 (0.11-54.97) | NA |

*p<0.10, **p<0.05, ***p<0.01

^a^All reported outcomes were not adjusted for confounders. Effect sizes in bold denote statistical significance. AEs were primarily classified according to the according to the Division of AIDS (DAIDS) Table for Grading the Severity of Adult and Pediatric Adverse Events, Corrected Version 2.1[70]. ^b^One of more studies included in the analysis only include patients with severe and/or serious adverse events. ^c^Overlapping populations were observed between Brust et al[41,61], Hong et al[42], Padayatchi et al[26], and Smith et al[34], of which Smith et al[34] was prioritized due to larger sample size. ^d^Four studies[20,55–57] were identified as one cohort due to same populations. ^e^Unpublished data, provided by request. ^f^Psychiatric disorders include anxiety, mental confusion, depression, hallucinations, hearing voices, psychosis, and convulsions/seizure. ^g^When both audiometric and clinical findings were available, audiometric findings were prioritized for analysis.

**AE,** adverse event; **ALP,** alkaline phosphatase; **ALT,** alanine aminotransferase; **AST,** aspartate aminotransferase; **CI,** confidence interval; **HIV,** human immunodeficiency virus; **I^2^,** I-squared heterogeneity value; **RR,** risk ratio.

**References**

1. VanderWeele TJ. On a square-root transformation of the odds ratio for a common outcome. Epidemiology. 2017;28: e58–e60. doi:10.1097/EDE.0000000000000733

2. Furuya-Kanamori L, Barendregt JJ, Doi SAR. A new improved graphical and quantitative method for detecting bias in meta-analysis. Int J Evid Based Healthc. 2018;16: 195–203. doi:10.1097/XEB.0000000000000141

3. Deeks JJ, Higgins JPT, Altman DG. Chapter 10: Analysing data and undertaking meta-analyses. 2nd ed. In: Higgins J, Thomas J, Chandler J, Cumpston M, Li T, Page M, et al., editors. Cochrane handbook for systematic reviews of interventions. 2nd ed. Chichester (UK): John Wiley & Sons; 2019.

4. Higgins J, Thomas J, Chandler J, Cumpston M, Li T, Page M, et al., editors. Cochrane handbook for systematic reviews of interventions version 6.0 (updated July 2019). 6th ed. Cochrane Handbook for Systematic Reviews of Interventions. Cochrane; 2019. doi:10.1002/9781119536604

5. Peters JL, Sutton AJ, Jones DR, Abrams KR, Rushton L. Contour-enhanced meta-analysis funnel plots help distinguish publication bias from other causes of asymmetry. J Clin Epidemiol. 2008;61: 991–996. doi:10.1016/j.jclinepi.2007.11.010

6. Egger M, Smith GD, Schneider M, Minder C. Bias in meta-analysis detected by a simple, graphical test. BMJ. 1997;315: 629 LP – 634. doi:10.1136/bmj.315.7109.629

7. Begg CB, Mazumdar M. Operating characteristics of a rank correlationtTest for publication bias. Biometrics. 1994;50: 1088–1101.

8. Duval S, Tweedie R. Trim and fill: A simple funnel-plot-based method of testing and adjusting for publication bias in meta-analysis. Biometrics. 2000;56: 455–463. doi:10.1111/j.0006-341x.2000.00455.x

9. R Core Team. R: A language and environment for statistical computing. Vienna: R Foundation for Statistical Computing; 2020.

10. Balduzzi S, Rücker G, Schwarzer G. How to perform a meta-analysis with R: a practical tutorial. Evid Based Ment Health. 2019;22: 153–160. doi:10.1136/ebmental-2019-300117

11. Viechtbauer W. Conducting meta-analyses in R with the metafor package. J Stat Softw. 2010;1.

12. McGuinness LA, Higgins JPT. Risk-of-bias visualization (robvis): An R package and Shiny web app for visualizing risk-of-bias assessments. Research Synthesis Methods. 2020. pp. 1–7. doi:10.1002/jrsm.1411

13. Barendregt JJ, Doi SA. MetaXL user guide, version 5.3. Queensland: EpiGear International Pty Ltd; 2015. pp. 1–52.

14. Riley RD, Moons KGM, Snell KIE, Ensor J, Hooft L, Altman DG, et al. A guide to systematic review and meta-analysis of prognostic factor studies. BMJ. 2019;364. doi:10.1136/bmj.k4597

15. Shibeshi W, Sheth AN, Admasu A, Berha AB, Negash Z, Yimer G. Nephrotoxicity and ototoxic symptoms of injectable second-line anti-tubercular drugs among patients treated for MDR-TB in Ethiopia: A retrospective cohort study. BMC Pharmacol Toxicol. 2019;20: 31. doi:10.1186/s40360-019-0313-y

16. Velingker A, Lawande D. Adverse drug reactions and treatment outcome analysis in multidrug resistant tuberculosis patients at a DOTS plus site. Int J Basic Clin Pharmacol. 2020;9: 547. doi:10.18203/2319-2003.ijbcp20201174

17. Lakhani P, Barua S, Singh D, Jain S, Kant S, Verma A, et al. An observational study to find out incidence and pattern of adverse drug reactions among multidrug resistant tuberculosis patients treated under revised national TB control program of India. Int J Basic Clin Pharmacol. 2019;8: 320. doi:10.18203/2319-2003.ijbcp20190154

18. Pym AS, Diacon AH, Tang SJ, Conradie F, Danilovits M, Chuchottaworn C, et al. Bedaquiline in the treatment of multidrug- and extensively drugresistant tuberculosis. Eur Respir J. 2016;47: 564–574. doi:10.1183/13993003.00724-2015

19. Jones J, Mudaly V, Voget J, Naledi T, Maartens G, Cohen K. Adverse drug reactions in South African patients receiving bedaquiline-containing tuberculosis treatment: An evaluation of spontaneously reported cases. BMC Infect Dis. 2019;19: 544. doi:10.1186/s12879-019-4197-7

20. Sagwa EL. Prevalence and risk factors of adverse events during treatment in of drug of resistant in tuberculosis setting virus immunodeficiency Namibia : 2009-10. University of the Western Cape. 2012.

21. Matambo R, Takarinda KC, Thekkur P, Sandy C, Mharakurwa S, Makoni T, et al. Treatment outcomes of multi drug resistant and rifampicin resistant Tuberculosis in Zimbabwe: A cohort analysis of patients initiated on treatment during 2010 to 2015. PLoS ONE. Public Library of Science; 2020. doi:10.1371/journal.pone.0230848

22. Jikijela O. Clinical characteristics and treatment outcomes of multi-drug resistant tuberculosis patients attending a hospital in Buffalo city metropolitan municipality, Eastern Cape. University of the Western Cape. 2018.

23. Ndjeka N, Schnippel K, Master I, Meintjes G, Maartens G, Romero R, et al. High treatment success rate for multidrug-resistant and extensively drug-resistant tuberculosis using a bedaquiline-containing treatment regimen. Eur Respir J. 2018;52: 1801528. doi:10.1183/13993003.01528-2018

24. Ndjeka N, Conradie F, Schnippel K, Hughes J, Bantubani N, Ferreira H, et al. Treatment of drug-resistant tuberculosis with bedaquiline in a high HIV prevalence setting: An interim cohort analysis. Int J Tuberc Lung Dis. 2015;19: 979–985. doi:10.5588/ijtld.14.0944

25. Olayanju O, Limberis J, Esmail A, Oelofse S, Gina P, Pietersen E, et al. Long-term bedaquiline-related treatment outcomes in patients with extensively drug-resistant tuberculosis from South Africa. Eur Respir J. 2018;51: 1800544. doi:10.1183/13993003.00544-2018

26. Padayatchi N, Gopal M, Naidoo R, Werner L, Naidoo K, Master I, et al. Clofazimine in the treatment of extensively drug-resistant tuberculosis with HIV coinfection in South Africa: A retrospective cohort study. J Antimicrob Chemother. 2014;69: 3103–3107. doi:10.1093/jac/dku235

27. Thee S, Garcia-Prats AJ, Draper HR, McIlleron HM, Wiesner L, Castel S, et al. Pharmacokinetics and safety of moxifloxacin in children with multidrug-resistant tuberculosis. Clin Infect Dis. 2015;60: 549–556. doi:10.1093/cid/ciu868

28. Solante M, Chagan-Yasutan H, Hattori T, Leano S, Garfin AMC, Soolingen D, et al. High rates of human immunodeficiency virus and drug resistance in tuberculosis patients in Manila, Philippines. Biomed Biotechnol Res J. 2017;1: 157. doi:10.4103/bbrj.bbrj_72_17

29. Seung KJ, Omatayo DB, Keshavjee S, Furin JJ, Farmer PE, Satti H. Early outcomes of MDR-TB treatment in a high HIV-prevalence setting in southern Africa. PLoS One. 2009;4. doi:10.1371/journal.pone.0007186

30. Kashongwe IM, Mawete F, Mbulula L, Nsuela DJ, Losenga L, Anshambi N, et al. Outcomes and adverse events of pre- And extensively drug-resistant tuberculosis patients in Kinshasa, Democratique Republic of the Congo: A retrospective cohort study. PLoS One. 2020;15. doi:10.1371/journal.pone.0236264

31. Magiorakos A-P, Srinivasan A, Carey RB, Carmeli Y, Falagas ME, Giske CG, et al. Multidrug-resistant, extensively drug-resistant and pandrug-resistant bacteria: an international expert proposal for interim standard definitions for acquired resistance. Clin Microbiol Infect Off Publ Eur Soc Clin Microbiol Infect Dis. 2012;18: 268–281. doi:10.1111/j.1469-0691.2011.03570.x

32. Singh A, Prasad R, Balasubramanian V, Gupta N. Drug-resistant tuberculosis and HIV infection: Current perspectives. HIV/AIDS (Auckland). 2020;12: 9–31. doi:10.2147/HIV.S193059

33. Higgins JPT, Thomas J, Chandler J, Cumpston M, Li T, Page MJ, et al., editors. Cochrane handbook for systematic reviews of interventions version 6.0 (updated July 2019). 6th ed. Cochrane Handbook for Systematic Reviews of Interventions. Cochrane; 2019. doi:10.1002/9781119536604

34. Smith JP, Gandhi NR, Shah NS, Mlisana K, Moodley P, Johnson BA, et al. The impact of concurrent antiretroviral therapy and MDR-TB treatment on adverse events. J Acquir Immune Defic Syndr. 2020;83: 47–55. doi:10.1097/QAI.0000000000002190

35. Conradie F, Diacon AH, Ngubane N, Howell P, Everitt D, Crook AM, et al. Bedaquiline, pretomanid and linezolid for treatment of extensively drug resistant, intolerant or non-responsive multidrug resistant pulmonary tuberculosis. N Engl J Med. 2020;382: 893–902. doi:10.1056/nejmoa1901814

36. Letswee G, Kamau H, Gaida R, Truter I. Haematological adverse effects associated with linezolid in patients with drug‐resistant tuberculosis: an exploratory study. Int J Pharm Pract. 2019;27: 575–577. doi:10.1111/ijpp.12543

37. Hughes J, Reuter A, Chabalala B, Isaakidis P, Cox H, Mohr E. Adverse events among people on delamanid for rifampicin-resistant tuberculosis in a high HIV prevalence setting. Int J Tuberc Lung Dis. 2019;23: 1017–1023. doi:10.5588/ijtld.18.0651

38. Merid MW, Gezie LD, Kassa GM, Muluneh AG, Akalu TY, Yenit MK. Incidence and predictors of major adverse drug events among drug-resistant tuberculosis patients on second-line anti-tuberculosis treatment in Amhara regional state public hospitals; Ethiopia: a retrospective cohort study. BMC Infect Dis. 2019;19: 286. doi:10.1186/s12879-019-3919-1

39. Olayanju O, Esmail A, Limberis J, Gina P, Dheda K. Linezolid interruption in patients with fluoroquinolone-resistant tuberculosis receiving a bedaquiline-based treatment regimen. Int J Infect Dis. 2019;85: 74–79. doi:10.1016/j.ijid.2019.04.028

40. Sineke T, Evans D, Schnippel K, van Aswegen H, Berhanu R, Musakwa N, et al. The impact of adverse events on health-related quality of life among patients receiving treatment for drug-resistant tuberculosis in Johannesburg, South Africa. Health Qual Life Outcomes. 2019;17: 94. doi:10.1186/s12955-019-1155-4

41. Brust JCM, Shah NS, Mlisana K, Moodley P, Allana S, Campbell A, et al. Improved survival and cure rates with concurrent treatment for multidrug-resistant tuberculosis-human immunodeficiency virus coinfection in South Africa. Clin Infect Dis. 2018;66: 1246–1253. doi:10.1093/cid/cix1125

42. Hong H. Risk of aminoglycoside-induced hearing loss among patients with drug-resistant tuberculosis in South Africa. 2018.

43. Perumal R, Abdelghani N, Naidu N, Yende-Zuma N, Dawood H, Naidoo K, et al. Risk of nephrotoxicity in patients with drug-resistant tuberculosis treated With kanamycin/capreomycin with or without concomitant use of tenofovir-containing antiretroviral therapy. J Acquir Immune Defic Syndr. 2018;78: 536–542. doi:10.1097/QAI.0000000000001705

44. Dela AI, Tank NKD, Singh AP, Piparva KG. Adverse drug reactions and treatment outcome analysis of DOTS-plus therapy of MDR-TB patients at district tuberculosis centre: A four year retrospective study. Lung India. 2017;34: 522–526. doi:10.4103/0970-2113.217569

45. Huerga H, Bastard M, Kamene M, Wanjala S, Arnold A, Oucho N, et al. Outcomes from the first multidrug-resistant tuberculosis programme in Kenya. Int J Tuberc Lung Dis. 2017;21: 314–319. doi:10.5588/ijtld.16.0661

46. Sagwa EL, Ruswa N, Mavhunga F, Rennie T, Mengistu A, Mekonen TT, et al. Renal function of MDR-TB patients treated with kanamycin regimens or concomitantly with antiretroviral agents. Int J Tuberc Lung Dis. 2017;21: 1245–1250. doi:10.5588/ijtld.16.0953

47. Kelly AM, Smith B, Luo Z, Given B, Wehrwein T, Master I, et al. Discordance between patient and clinician reports of adverse reactions to MDR-TB treatment. Int J Tuberc Lung Dis. 2016;20: 442–447. doi:10.5588/ijtld.15.0318

48. Mehta S, Das M, Laxmeshwar C, Jonckheere S, Thi SS, Isaakidis P. Linezolid-associated optic neuropathy in drug-resistant tuberculosis patients in Mumbai, India. PLoS One. 2016;11. doi:10.1371/journal.pone.0162138

49. Schnippel K, Berhanu RH, Black A, Firnhaber C, Maitisa N, Evans D, et al. Severe adverse events during second-line tuberculosis treatment in the context of high HIV Co-infection in South Africa: A retrospective cohort study. BMC Infect Dis. 2016;16. doi:10.1186/s12879-016-1933-0

50. Sogebi OA, Adefuye BO, Adebola SO, Oladeji SM, Adedeji TO. Clinical predictors of aminoglycoside-induced ototoxicity in drug-resistant tuberculosis patients on intensive therapy. Auris Nasus Larynx. 2017;44: 404–410. doi:10.1016/j.anl.2016.10.005

51. Avong YK, Isaakidis P, Hinderaker SG, Van Den Bergh R, Ali E, Obembe BO, et al. Doing no harm? Adverse events in a nation-wide cohort of patients with multidrug-resistant tuberculosis in Nigeria. PLoS One. 2015;10. doi:10.1371/journal.pone.0120161

52. Meressa D, Hurtado RM, Andrews JR, Diro E, Abato K, Daniel T, et al. Achieving high treatment success for multidrug-resistant TB in Africa: Initiation and scale-up of MDR TB care in Ethiopia - An observational cohort study. Thorax. 2015;70: 1181–1188. doi:10.1136/thoraxjnl-2015-207374

53. Modongo C, Pasipanodya JG, Zetola NM, Williams SM, Sirugo G, Gumboc T. Amikacin concentrations predictive of ototoxicity in multidrug-resistant tuberculosis patients. Antimicrob Agents Chemother. 2015;59: 6337–6343. doi:10.1128/AAC.01050-15

54. Sagwa EL, Ruswa N, Mavhunga F, Rennie T, Leufkens HGM, Mantel-Teeuwisse AK. Comparing amikacin and kanamycin-induced hearing loss in multidrug-resistant tuberculosis treatment under programmatic conditions in a Namibian retrospective cohort. BMC Pharmacol Toxicol. 2015;16: 36. doi:10.1186/s40360-015-0036-7

55. Sagwa E, Ruswa N, Musasa JP, Mantel-Teeuwisse AK. Adverse events during treatment of drug-resistant tuberculosis: A comparison between patients with or without human immunodeficiency virus co-infection. Drug Saf. 2013;36: 1087–1096. doi:10.1007/s40264-013-0091-1

56. Sagwa EL, Mantel-Teeuwisse AK, Ruswa NC. Occurrence and clinical management of moderate-to-severe adverse events during drug-resistant tuberculosis treatment: a retrospective cohort study. J Pharm Policy Pract. 2014;7. doi:10.1186/2052-3211-7-14

57. Sagwa E, Mantel-Teeuwisse AK, Ruswa N, Musasa JP, Pal S, Dhliwayo P, et al. The burden of adverse events during treatment of drug-resistant tuberculosis in Namibia. South Med Rev. 2012;5: 6–13.

58. Charles M, Vilbrun SC, Koenig SP, Hashiguchi LM, Mabou MM, Ocheretina O, et al. Treatment outcomes for patients with multidrug-resistant tuberculosis in post-earthquake Port-au-Prince, Haiti. Am J Trop Med Hyg. 2014;91: 715–721. doi:10.4269/ajtmh.14-0161

59. Conradie F, Mabiletsa T, Sefoka M, Mabaso S, Louw R, Evans D, et al. Prevalence and incidence of symmetrical symptomatic peripheral neuropathy in patients with multidrug-resistant TB. South African Med J. 2014;104: 24–26. doi:10.7196/SAMJ.6455

60. Modongo C, Sobota RS, Kesenogile B, Ncube R, Sirugo G, Williams SM, et al. Successful MDR-TB treatment regimens including Amikacin are associated with high rates of hearing loss. BMC Infect Dis. 2014;14. doi:10.1186/1471-2334-14-542

61. Brust JCM, Shah NS, Van Der Merwe TL, Bamber S, Ning Y, Heo M, et al. Adverse events in an integrated home-based treatment program for MDR-TB and HIV in Kwazulu-Natal, South Africa. J Acquir Immune Defic Syndr. 2013;62: 436–440. doi:10.1097/QAI.0b013e31828175ed

62. Seddon JA, Thee S, Jacobs K, Ebrahim A, Hesseling AC, Schaaf HS. Hearing loss in children treated for multidrug-resistant tuberculosis. J Infect. 2013;66: 320–329. doi:10.1016/j.jinf.2012.09.002

63. Shean K, Streicher E, Pieterson E, Symons G, van Zyl Smit R, Theron G, et al. Drug-associated adverse events and their relationship with outcomes in patients receiving treatment for extensively drug-resistant tuberculosis in South Africa. PLoS One. 2013;8. doi:10.1371/journal.pone.0063057

64. Van der Walt M, Lancaster J, Odendaal R, Davis JG, Shean K, Farley J. Serious treatment related adverse drug reactions amongst anti-retroviral naïve MDR-TB patients. PLoS One. 2013;8: e58817. doi:10.1371/journal.pone.0058817

65. Harris T, Bardien S, Schaaf HS, Petersen L, de Jong G, Fagan JJ, et al. Aminoglycoside-induced hearing loss in HIV-positive and HIV-negative multidrug-resistant tuberculosis patients. South African Med J. 102.

66. Jacobs T, Ross A. Adverse effects profile of multidrug-resistant tuberculosis treatment in a South African outpatient clinic. South African Fam Pract. 2012;54: 531–539. doi:10.1080/20786204.2012.10874288

67. Kvasnovsky CL, Cegielski JP, Erasmus R, Siwisa NO, Thomas K, der Walt ML van. Extensively drug-resistant TB in Eastern Cape, South Africa: High mortality in HIV-negative and HIV-positive patients. JAIDS J Acquir Immune Defic Syndr. 2011;57: 146–152. doi:10.1097/QAI.0b013e31821190a3

68. Burgos M, Gonzalez LC, Paz EA, Gournis E, Kawamura LM, Schecter G, et al. Treatment of multidrug‐resistant tuberculosis in San Francisco: An outpatient‐based approach. Clin Infect Dis. 2005;40: 968–975. doi:10.1086/428582

69. Viera AJ. Odds ratios and risk ratios: what’s the difference and why does it matter? South Med J. 2008;101: 730–734. doi:10.1097/SMJ.0b013e31817a7ee4

70. National Institute of Allergy and Infectious Diseases. Division of AIDS (DAIDS) table for grading the severity of adult and pediatric adverse events, corrected version 2.1. U.S. Department of Health and Human Services, National Institutes of Health, National Institute of Allergy and Infectious Diseases, Division of AIDS; 2017. doi:10.7326/0003-4819-157-11-201212040-00003
