# Supplementary material for: The effect of human immunodeficiency virus infection on adverse events during treatment of drug-resistant tuberculosis: A systematic review and meta-analysis
Source: PLoS One. 2021 Mar 4;16(3):e0248017. doi: 10.1371/journal.pone.0248017 (PMC7932087; doi:10.1371/journal.pone.0248017)
Supplement: Attachment — Submitted filename: [FINAL] Point by Point Responses_PLoS One_R2.docx [file pone.0248017.s004.docx]

February 16th, 2021

Dear Prof. Joerg Heber, Dr. Endi Lanza Galvão, and respected reviewers,

Thank you for the comments from the editor and reviewers on our manuscript entitled “**The effect of human immunodeficiency virus infection on adverse events during treatment of drug-resistant tuberculosis: a systematic review and meta-analysis”** by Gilbert Lazarus, Kevin Tjoa, Anthony William Brian Iskandar, Melva Louisa, Evans L. Sagwa, Nesri Padayatchi, Vivian Soetikno (manuscript ID: **PONE-D-20-36024**). We really appreciate the constructive and detailed feedback on our manuscript. We have revised the current submitted manuscript based on the reviewers’ feedback.

**Details of revisions**

| **No** | **Comments and recommendations** | **Revisions** |
| --- | --- | --- |
| **Reviewer 1** | | |
| 1 | It is stated in the response from authors that "the role of specific regimen and drug-drug interaction may provide essential information in determining which DR-TB treatment regimen to use. However, as we did not intend to analyze patient-level data, and as the treatment regimens used in each study intertwined with each other, we were unable to analyze specific drug-drug interaction". It would be good to include this in the section on limitations. | Thank you very much for the feedback. We agree with the reviewer that the fact that we only pooled aggregate-level data may imply that some potentially important factors remained unexplored in our study. In this regard, we have added the aforementioned fact as our limitation.  This study has several limitations. Although our findings highlighted the unfavorable effect of HIV co-infection on AE development in patients receiving DR-TB treatments, some of the pooled estimates were unadjusted for confounders due to study scarcity, emphasizing caution in the interpretation of our findings. In addition, there is a need for further studies evaluating the independent effect of ART and CD4 count on AE occurrence in HIV/DR-TB patients, and studies comparing the effect of HIV infection across different spectrum of AE seriousness and severity. We were also unable to perform subgroup analysis by duration of follow-up due to heterogeneity in reporting, suggesting that a standardized reporting of follow-up duration in future studies are urgently needed. Furthermore, our study was also limited by the fact that some potentially important factors such as specific ARV/anti-DR-TB regimens and drug-drug interactions remained unexplored as patient-level data were not available. [page 17 line 357-359] |

We hope you will kindly reconsider our submission and we are looking forward to the publication of our manuscript.

Thank you very much in advance for your kind consideration.

Sincerely yours,

Representing all authors

**Gilbert Lazarus**

Faculty of Medicine Universitas Indonesia,

Jl. Salemba Raya No. 6, Jakarta 10430, Indonesia,

*E-mail:* [*gilbert.lazarus@ui.ac.id*](mailto:gilbert.lazarus@ui.ac.id?subject=Query%20on%20Article%20Entitled%20%22The%20effect%20of%20human%20immunodeficiency%20virus%20infection%20on%20adverse%20events%20during%20treatment%20of%20drug-resistant%20tuberculosis:%20a%20systematic%20review%20and%20meta-analysis)
